# The Effect of Shear on the Properties of an Associated Polymer Solution for Oil Displacement

**DOI:** 10.3390/polym15030616

**Published:** 2023-01-25

**Authors:** Guoying Jiao, Shijie Zhu, Zhongbin Ye, Zheng Shu, Xijin Wang, Daming Wang

**Affiliations:** 1 Institute of Petroleum and Natural Gas Engineering, Chongqing University of Science and Technology, Chongqing 401331, China; 2 Chengdu Technological University, Chengdu 610031, China; 3 State Key Laboratory of Oil & Gas Reservoir and Exploitation Engineering, Southwest Petroleum University, Chengdu 610500, China; 4 Sichuan Energy Investment Oil and Gas Exploration and Development Co., Ltd., Chengdu 610095, China; 5 Shengli Oil Plant, Dongying 257051, China

**Keywords:** polymer, shear, rheology, viscoelasticity, adsorption retention, hydrodynamic dimensions

## Abstract

Polymer flooding is one of the techniques used to enhance oil recovery from depleted hydrocarbon reservoirs. Although this technology is popular for this application, the shearing effect in the injection process causes poor performance, which is an obstacle to meeting the needs of the formation. An experimental evaluation of the rheological properties, viscoelasticity, hydrodynamic size, static adsorption, and seepage characteristics of the associated polymer solution before and after shearing was conducted to determine the influence of shearing on the polymer solution. The results show that the effect of shear on the polymer was irreversible, and the properties of the polymer solution damaged by shear were attenuated. After the critical associating concentration, the associated polymer can recover its solution properties through hydrophobic association, which can improve the shear resistance of the polymer solution and make its own rheological law and reduce the viscoelastic change. Although the hydrodynamic size, viscoelasticity, and adsorption capacity of the polymer solution after shear failure decreased, strong flow resistance during porous media seepage and mobility control was achieved. Improving the shear resistance of the polymer solution by increasing the intermolecular force is proposed to develop new polymer systems for subsequent oil displacement.

## 1. Introduction

The principle of polymer flooding technology is to improve the oil displacement efficiency by increasing the viscosity of the displacement phase, reducing the seepage capacity of the displacement phase, and controlling mobility [1,2,3]. Such action mechanisms make polymer flooding technology the main tool to improve oil recovery in the later stages of water-driven development. By 2022, polymer flooding technology had been under development in China for over 70 years and is widely applied in major oilfields [3,4]. Although polymer flooding technology has achieved remarkable success, this technology has problems that need to be solved urgently. These obstacles hinder the further application and development of polymer flooding technology. One of the most critical problems in practical applications is the strong shearing effect of the polymer solution for oil displacement arising from, for example, shearing at the polymer injection pump, pipe bend, perforating tunnel, etc. The solution performance is significantly reduced under the action of strong mechanical shear, resulting in a significant difference between the underground polymer and ground preparation systems [5]. 

To cope with the inevitable mechanical shear effect in the application of polymer flooding and improve the performance of polymer solutions, the development of shear-resistant polymers is a popular research topic in polymer flooding [6,7]. Among these polymers, associative polymers have been widely studied [8,9,10]. Associative polymers are hydrophilic macromolecular chains with hydrophobic groups, the content of which is generally 2–5 mol%. Hydrophobic groups on the molecular chain cooperate with the electrostatic repulsion and attraction of charged-ion groups to make macromolecular chains produce intramolecular or intermolecular associations, combine to form independent aggregates, and form various supramolecular spatial network structures [11,12,13]. After the “dynamic physical glue network structure” formed by hydrophobic association is destroyed by external forces, this structure reassociates and restores part of the spatial structure [14,15]. This restored polymer exhibits good shear resistance, leading to widespread use. 

China’s Bohai Oilfield has optimized AP-P4 associative polymer as an oil displacement agent, and its polymer flooding performance is obviously better than that of polymer HPAM. Although the effect of shear on the polymer was fully considered in the process of polymer development, there are still some deficiencies in the related understanding. For example, how do the rheology, viscoelasticity, and adsorption capacity of the polymer solution change after mechanical shear failure, and how many solution properties remain [16]. It will be beneficial to the application of polymer for oil displacement to carry out analysis and research on the shear effect of polymers for oil displacement based on the existing research methods centered on the influence of mechanical shear on polymer solution.

In this study, the industrial application of polymer systems is considered. By analyzing the changes in polymer solution properties before and after shearing, the effects of shearing on the viscoelasticity, rheology, and other properties of existing polymer injection system solutions were explored, providing a new direction for the development of polymer synthesis technology that supports the progress of polymer flooding technology.

## 2. Experiment

### 2.1. Experimental Materials and Equipment

**Figure 1:**
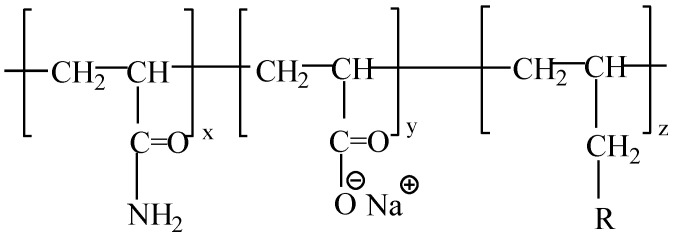
The molecular formula of AP-P4.

(1) Experimental polymer [17]: AP-P4 (Sichuan Guangya Company, Nanchong, China) is a new water-soluble polymer formed by introducing a small number of hydrophobic groups (0.8 mol%) into the hydrophilic main chain of partially hydrolyzed polyacrylamide. Its molecular structure contains three types of monomers: main monomers (acrylamide and acrylic acid) and hydrophobic-association monomers. The molecular weight, solid contents, and degree of hydrolysis were 18 million, 88%, and 23.6%, respectively. The molecular structure of this polymer is shown in Figure 1. The polymer of this system has strong solution properties and can maintain certain solution properties under strong shear by analyzing its shear action.

(2) Experimental brine: 3000 mg/L sodium chloride was used to simulate the influence of cations in the aqueous solution on the polymer solution. (The higher the salinity, the greater the performance of the polymer solution. The determination of a specific concentration of salinity is the basis of the study. The selection of low salinity is conducive to the analysis of the impact of shear action on the polymer solution.)

(3) Experimental core: φ 25 × 800 mm, the average porosity was about 32%, and the permeability was 2500 mD.

(4) Experimental temperature: 20 °C. 

(5) Experimental instruments: RS600 rotary rheometer (Haake Technik GmbH, Vreden, Germany), mechanical stirrer (IKA-Werke, Staufen, Germany), electronic balance (Shanghai Shunyu Hengping Scientific Instrument Co., Ltd., Shanghai, China), UV-8000 ultraviolet spectrophotometer (Shanghai Precision Instrument Co., Ltd., Shanghai, China), ISCO pump (Teledyne ISCO, Lincoln, NE, USA), volumetric flask, intermediate container, membrane holder, and microporous membrane (Chengdu Rock Core Technology Co., Ltd., Chengdu, China), and pressure sensor (Guangzhou Senas Instrument Co., Ltd., Guangzhou, China), Waring 2000B stirrer (Beijing Zhongxi Huada Technology Co., Ltd., Beijing, China).

### 2.2. Experimental Information and Procedures 

#### 2.2.1. Rheological/Viscoelastic Determination of Polymer Solutions

The critical association concentration of AP-P4 is 1400 mg/L [18]. Because of the high viscosity-increasing property of the associating polymer, the solution properties of the associating polymer are obviously different before and after the critical associating concentration. Therefore, the influence of this association should be considered in subsequent research. Furthermore, 1000 mg/L and 2000 mg/L concentrations were selected to prepare the AP-P4 polymer solution. The polymer solution was prepared in the sampling section using a Waring stirrer to simulate different shear strengths of 5 s, 20 s, and 40 s and then was kept on standby after defoaming (First gear = Speed: 3500 rpm) [18,19]. An RS600 rheometer was used to test the rheology and viscoelasticity of the polymer solution after shearing and without shearing. As pointed out by [20,21,22], non-Newtonian fluids, such as viscoelastic fluids, in turbulent flow produce extra stresses, due to the correlation between the fluctuation in the apparent viscosity and the shear rate, which can alter the outcome of the measurement. it is important to keep a low Reynolds/Taylor number regime, according to the above-mentioned references. (1) An RS600 rheometer (plate-cone) was used to determine the rheological characteristics of the polymer solutions. The CR rotation step was adopted with a shear rate set from λ = 0.01 s^−1^ to λ = 1300 s^−1^, while a dual-barrel test system and dg41ti rotor were used in the test. (2) An RS600 rheometer (plate-cone) was used to determine the viscoelastic characteristics of the polymer solutions, with angular frequencies from 10 rad/s to 0.01 rad/s and step lengths of 4.

#### 2.2.2. Hydrodynamic Dimension Test and Analysis

The hydrodynamic radius of the polymer solution measured using the microporous filter membrane method is not the hydrodynamic radius of a single polymer molecule. This measurement reflects the hydrodynamic characteristic size of the polymer solution and the ability of the polymer solution to diffuse through pores of a specific size under specific conditions [22]. The measurement method was as follows. First, a polymer solution with a target concentration of 2000 mg/L was prepared, its apparent viscosity was tested, and then the solution was placed in an intermediate container. The microporous membrane was then placed in a membrane holder. The polymer solution was added to the intermediate container by adjusting the pressure of the nitrogen cylinder to control the pressure at 0.3 MPa. Finally, a polymer with a fixed liquid volume was taken from the measuring cylinder, and the viscosity of the filtrate was tested using the RS600 rheometer to determine the hydrodynamic size. Similarly, first gear shear conditions of 20 s and 40 s were selected, and then the hydrodynamic dimensions were tested according to the above method.

#### 2.2.3. Polymer Static Adsorption Capacity Test

Polymer solutions of 100 mg/L, 250 mg/L, 500 mg/L, 750 mg/L, 800 mg/L, 1000 mg/L, 1400 mg/L, 1800 mg/L, 2000 mg/L, and 2500 mg/L were prepared. An 80 mL polymer solution was added to a wide-mouth bottle, while the rest of the liquid was sheared with a Waring stirrer for 20 s, and then the 80 mL polymer solution was measured in the wide-mouth bottle. Next, acid-washed quartz sand was added in a solid-liquid ratio of 1:9, stirred, and mixed. After full contact for 24 h, the clear upper solution was collected, and its solution concentration was measured with an ultraviolet spectrophotometer [23]. The standard curve formula for the relationship between the AP-P4 polymer solution concentration and absorbance is y = 0.0191x − 0.3031, R = 0.9875 [24].

The static adsorption capacity of the polymer solution on the surface of the quartz sand was calculated using Equation (1).

(1) $$\Gamma =\frac{V\left(C_{0}-C_{e}\right)}{q}$$

where *Γ* is the adsorption amount, which represents the amount of polymer adsorbed per gram of rock particles (μg/g); *V* is the volume of the polymer solution (mL); *C*_0_ is the initial concentration of the polymer solution (mg/L); *C_e_* is the concentration of the polymer solution after adsorption equilibrium (mg/L); and *q* is the mass of quartz sand particles (g).

#### 2.2.4. Percolation Characteristics of the Polymer Solution

The specific experimental steps are as follows [25]. (1) The process was connected, the target solution was added to an intermediate container of polymer and experimental brine, and the solution was drained. (2) Saturated water was evacuated from the core, test brine was injected into the core at a flow rate of 1 mL/min, the permeability was measured, and the stable pressure was recorded. (3) The polymer solution was injected at a concentration of 2000 mg/L and rate of 1 mL/min (the solution without shear and solution after 20 s shear in the first gear), a sample was removed from the outlet every other time, and the mass of the sampled polymer was weighed; the polymer concentration was measured until the concentration of the polymer solution at the outlet end was equal to the polymer concentration at the inlet end. After the injection pressure stabilized, the injection pressure was recorded and the experiment ended. (4) The concentration of the sampled solution was measured with an ultraviolet spectrophotometer. According to the change in the concentration value, the amount of the polymer retained in the rock core was calculated based on a material balance.

(2) $$R=\frac{C_{0}V_{0}-\sum_{i=1}^{n}C_{i}V_{i}}{W}$$

where *R* is the retention of polymer in the rock core (μg/g sand), *C*_0_ is the inlet concentration of the polymer solution (mg/mL), *C_i_* is the outlet concentration of the polymer solution (mg/mL), *V*_0_ is the volume of the injected polymer solution (mL), *V_i_* is the outlet volume of the polymer solution (mL), and W is the dry weight of the core (g).

## 3. Results and Discussion

### 3.1. An Analysis of the Shear Effect on the Viscoelasticity of Polymer Solutions

**Figure 2:**
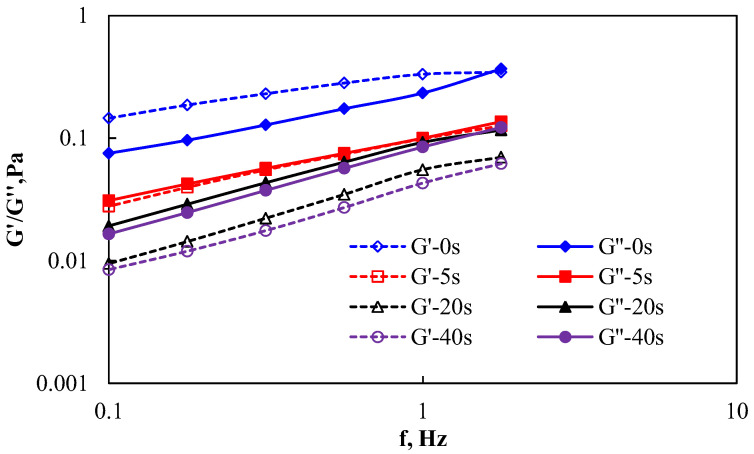
Effect of shear on the viscoelasticity of AP-P4 at a concentration of 1000 mg/L.

**Figure 3:**
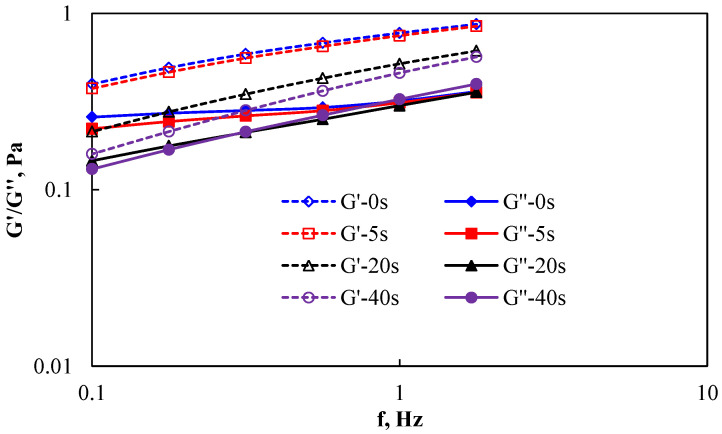
Effect of shear on the viscoelasticity of AP-P4 at a concentration of 2000 mg/L.

The experimental results of the effect of shear on the viscoelasticity of AP-P4 are shown in Figure 2 and Figure 3 (G’-Storage modulus; G’’-loss modulus).

At a low concentration (i.e. below the critical association concentration) of AP-P4, the polymer solution did not undergo intermolecular association, as shown in Figure 2. The shear action produced an obvious shear failure, and the viscosity and elastic moduli decreased significantly. The decrease in the viscosity modulus was relatively low, and the decrease in the elastic modulus was significant. The analysis shows that the intermolecular interaction force was weak; a stronger shearing force is associated with more damage to the molecular structure and a larger impact on its elastic action characteristics. It can be speculated that the performance of the system will be greatly reduced after the low-concentration polymer encounters mechanical shear during the injection process, and it is difficult to establish the flow resistance through its own viscoelasticity under the reservoir conditions to achieve the improvement in sweep and oil displacement efficiency.

As shown in Figure 3, 2000 mg/L polymer solution greatly enhanced the intermolecular force, clarifying the elastic action characteristics of the system and showing that the elastic modulus plays a dominant role. Although the storage modulus and dissipation modulus decreased with increasing shear strength, the elastic modulus after shear failure was higher than the viscous modulus. This result shows that the associated polymer exhibited completely different viscoelastic behavior after association than before association, entangling the molecular groups after shear damage, restoring part of the structural characteristics, and maintaining a certain elastic deformation capacity of the system [26]. This result also shows that, although the polymer solution injected into the formation was subject to shear failure, the elastic deformation of the system still produced greater elastic viscosity at the small pore throat when passing through the porous medium; thus, mobility control and a better oil displacement effect were achieved [27].

Comparing the polymer AP-P4 at the above two concentrations, it can be seen that the association effect is very important for improving the performance of the polymer solution, lending it stronger shear resistance. As long as the concentration of polymer solution can promote the association between each other, it can maintain certain system performance and realize the control of mobility.

### 3.2. The Effect of Shear on the Rheological Law of a Polymer Solution

**Figure 4:**
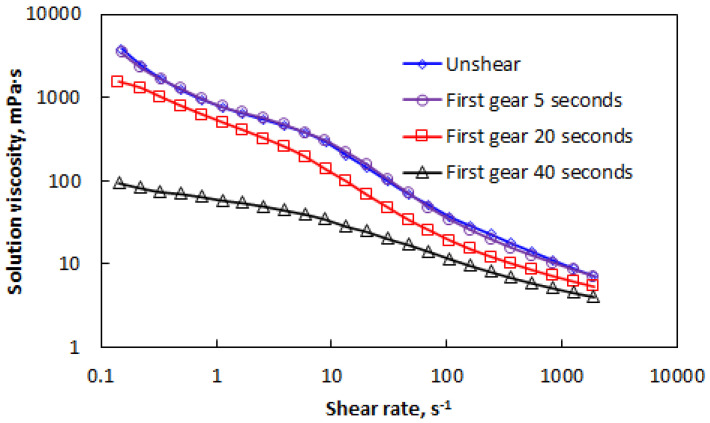
Effect of shear on the rheological curve of AP-P4 at a concentration of 1000 mg/L.

The rheological curves of AP-P4 at a solution concentration of 1000 mg/L and different shear strengths are shown in Figure 4.

As shown in Figure 4, the 1000 mg/L AP-P4 rheological curve decreased gradually with increasing shear rate, but the four rheological curves were significantly different. The association of AP-P4 occurred before the critical association concentration, which enhances the chain stretching, orientation, and entanglement/de-entanglement forces. However, mechanical destruction of the molecular chain by shearing destroys the intermolecular bonding force of the polymer. A stronger degree of mechanical shear damage is associated with a greater impact on intramolecular binding. This result explains the minimal difference between the curve characteristics of samples sheared for 5 s and 20 s, whereas the curve of 40 s of shearing is obviously different.

**Figure 5:**
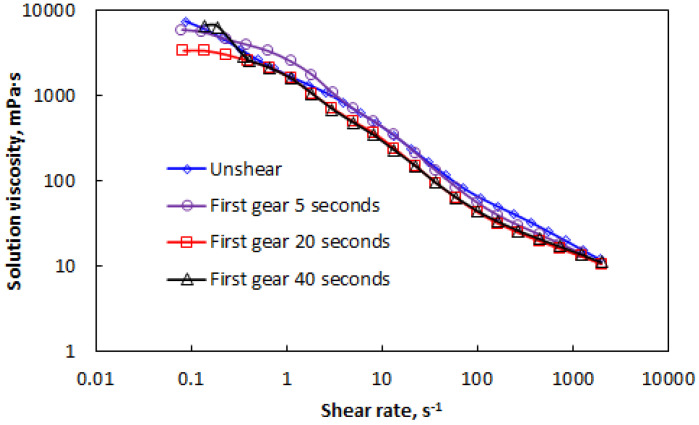
Effect of shear on the rheological curve of AP-P4 with a concentration of 2000 mg/L.

For AP-P4 with a solution concentration of 2000 mg/L, the rheological curves under different shear strengths are shown in Figure 5.

As shown in Figure 5, the four rheological curves exhibited strong regularity and high consistency. The spatial aggregation behavior formed by the association completely changed the viscosity-increasing mode of the polymer. The structural viscosity formed by intermolecular associations plays a dominant role in the viscosity variation of the solution. The molecular chains destroyed by shear exhibit spatial aggregation behavior, which dominates the characteristics of the shear rheological curve. However, the molecular chains destroyed by mechanical shear will reduce the association ability, which shows that increased shear strength is associated with decreased apparent viscosity. This result also agrees with the general rule of action of mechanical shear on the polymer solution.

Compared with the stress effect of the polymer at two concentrations, the stress effect experienced by polymer solution with high concentration at the same shear rate is greater. It is believed that the strong internal force of high-concentration polymer solution brings strong resistance, resulting in a more obvious increase in shear stress.

### 3.3. The Effect of Shear on the Hydrodynamic Size of the Polymer Solution 

**Figure 6:**
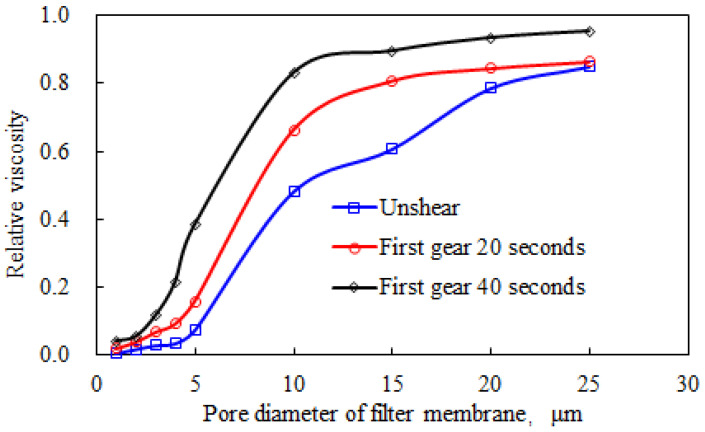
Effect of Shear on AP-P4 Hydrodynamic Size.

The experimental results for the effect of shear on the hydrodynamic size of the polymer solution at a concentration of 2000 mg/L are shown in Figure 6. (Relative viscosity refers to the ratio of the viscosity of the solution passing through the filter screen membrane to the viscosity of the solution not passing through the filter screen).

The hydrodynamic size of AP-P4 (see Figure 6) decreased slightly under the action of mechanical shear. The inflection point of the relative viscosity of the polymer solution before and after shearing remained at 10 μm, which indicates that the main hydrodynamic dimensions were relatively close. However, after shearing passes through 10 μm pores, the solution amount increased significantly, and 80% of the liquid traversed the pores during the first 40 s of shearing, which was 40% higher than that of the polymer solution without shearing. This result indicates that the hydrodynamic size of AP-P4 was reduced by shearing.

The decrease in hydrodynamic size will greatly affect the migration characteristics of polymer solution in porous media. Reduce the elastic triggering of polymer solution through porous media and the elastic viscosity after triggering. This will affect the effective viscosity of polymer solution in porous media, and ultimately reduce the control ability of polymer solution fluidity. The analysis of hydrodynamic dimensions further shows the negative effect of shear on polymer solution, and also shows that polymer AP-P4 has good shear resistance.

### 3.4. Analysis of the Effect of Shear on the Adsorption Capacity of Polymer Solution

The experimental results for the effect of shear on the static adsorption capacity of the polymer are presented in Table 1.

As shown in Table 1, the polymer solution before and after shearing conformed to Langmuir adsorption, but the adsorption amount of the polymer solution on the surface of the porous media decreased after shearing. The analysis shows that the shearing action destroyed the polymer molecular chain, which not only reduced the cohesion of the polymer but also reduced its adsorption thickness; thus, the original adsorption balance on the surface of quartz sand was broken, and the adsorption capacity was reduced. This result indicates that shear affects the adsorption capacity of the polymer solution on the surface of the medium. In addition, shear affects the adsorption and retention of polymers in porous media, reduces their residual resistance coefficient, and reduces their mobility control. However, the decrease in polymer adsorption also shows that the loss of solution concentration is relatively reduced and the effective application concentration is relatively high. We can make full use of this feature in engineering applications, reasonably design polymer injection parameters, and maximize the benefits of this effect.

### 3.5. Analysis of the Influence of Shear on the Percolation Characteristics of the Polymer Solution

**Figure 7:**
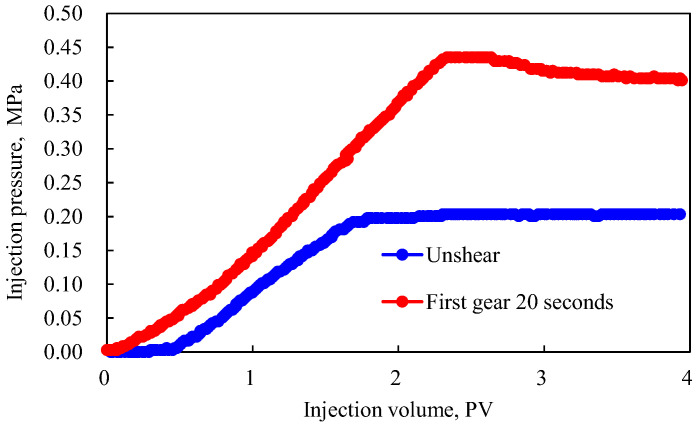
Effect of shear on the percolation characteristics of the polymer solution.

Based on the previous research, it was found that the viscoelasticity/rheology of a polymer solution after shearing would be significantly reduced, the hydrodynamic size would be reduced to a certain extent, and the adsorption capacity would be reduced. How does the polymer solution behave in porous media? As shown in Figure 7, the experimental results of polymer AP-P4 injected into porous media after shearing in the first gear of a Waring stirrer for 20 s are compared with the experimental results without shearing.

When AP-P4 was not sheared, strong viscoelastic properties under association were observed. Influenced by the pore throat conditions, the porous medium seepage exhibited complex viscoelastic characteristics and a complex solid-liquid interface interaction relationship, resulting in the polymer solution not reaching the injection equilibrium. However, after shearing, the molecular structure was destroyed, mobility control was reduced, and the speed of achieving injection balance accelerated. Although the ability to establish mobility resistance decreased, good mobility control was maintained. Compared with hydrolyzed polyacrylamide (HPAM), AP-P4 was less affected by shear [28].

On the one hand, the flow resistance of the polymer solution in porous media is affected by the internal resistance of polymer viscoelasticity; on the other hand, the interaction between the polymer and porous media also affects the flow resistance. Thus, the dynamic retention of the polymer solution before and after shearing was compared, and the experimental results are listed in Table 2.

An associative polymer solution has high adsorption and retention capacity in porous media, which is several times higher than that of ordinary polymers [28,29]. This fact also explains an aspect of its ability to establish higher mobility control. After shearing, the dynamic retention of AP-P4 decreased. This decrease occurred because the apparent viscosity, viscoelastic effect, and hydrodynamic size of the polymer system all decreased, hindering effective retention at the small pore throat. In addition, the adsorption capacity decreased with a decrease in the solution performance. Overall, the associated polymer can still establish good flow resistance and achieve mobility control after experiencing shear.

## 4. Conclusions

Associative polymers can recover part of their solution properties through hydrophobic association, which can improve the shear resistance of polymer solutions; thus, their rheological laws and viscoelastic changes do not attenuate significantly, and the negative effects of shear are reduced. Although the associated polymer after shear failure exhibited decreases in its hydrodynamic size, viscoelasticity, and adsorption capacity, flow resistance in porous media was established to achieve mobility control. Therefore, improving the shear resistance of a polymer solution by increasing the intermolecular force is a promising research topic of polymer systems for subsequent oil displacement.

## Figures and Tables

**Table 1:** Effect of Shear on the Static Adsorption Capacity of the Polymer.

**Adsorption Capacity, μg/g**	**Polymer Solution Concentration, mg/L**									
	100	250	500	750	800	1000	1400	1800	2000	2500
Unshear polymer	36.2	76.5	131.2	168.2	180.2	208.2	374.1	669.2	781	772.5
First gear 5 s	32.1	67.3	122.6	154.7	165.9	195.8	343.2	623.5	693.1	728.4
First gear 20 s	28.4	58.3	112.2	131.6	142.6	176.4	321.3	585.1	634.2	648.4
First gear 40 s	17.2	39.4	63.6	79.3	85.2	97.4	215.6	348.9	376.4	389.5

**Table 2:** Effect of Shear on the Dynamic Retention of the Polymer.

Shear State	Core Parameters		Dynamic Retention μg/g
	Permeability, mD	Porosity, %	
Unsheared polymer	2501	30.3	434.6
First gear 20 s	2457	30.3	245.2

## Data Availability

The data supporting the findings of this study are available from the corresponding author upon reasonable request.
